# Dynamic network modeling of gut microbiota during Alzheimer’s disease progression in mice

**DOI:** 10.1080/19490976.2023.2172672

**Published:** 2023-02-01

**Authors:** Yinhu Li, Yijing Chen, Yingying Fan, Yuewen Chen, Yu Chen

**Affiliations:** a Chinese Academy of Sciences Key Laboratory of Brain Connectome and Manipulation, Shenzhen Key Laboratory of Translational Research for Brain Diseases, the Brain Cognition and Brain Disease Institute, Shenzhen Institute of Advanced Technology, Chinese Academy of Sciences, Shenzhen–Hong Kong Institute of Brain Science–Shenzhen Fundamental Research Institutions, Shenzhen, China; b Guangdong Provincial Key Laboratory of Brain Science, Disease and Drug Development, HKUST Shenzhen Research Institute, Shenzhen, China

**Keywords:** Alzheimer’s disease, complex networks, gut microbiota, computational simulation, window period, hub bacteria

## Abstract

The intimate association between the gut microbiota (GM) and the central nervous system points to potential intervention strategies for neurological diseases. Nevertheless, there is currently no theoretical framework for selecting the window period and target bacteria for GM interventions owing to the complexity of the gut microecosystem. In this study, we constructed a complex network-based modeling approach to evaluate the topological features of the GM and infer the window period and bacterial candidates for GM interventions. We used Alzheimer’s disease (AD) as an example and traced the GM dynamic changes in AD and wild-type mice at one, two, three, six, and nine months of age. The results revealed alterations of the topological features of the GM from a scale-free network into a random network during AD progression, indicating severe GM disequilibrium at the late stage of AD. Through stability and vulnerability assessments of the GM networks, we identified the third month after birth as the optimal window period for GM interventions in AD mice. Further computational simulations and robustness evaluations determined that the hub bacteria were potential candidates for GM interventions. Moreover, our GM functional analysis suggested that *Lachnospiraceae* UCG-001 – the hub and enriched bacterium in AD mice – was the keystone bacterium for GM interventions owing to its contributions to quinolinic acid synthesis. In conclusion, this study established a complex network-based modeling approach as a practical strategy for disease interventions from the perspective of the gut microecosystem.

## Introduction

The gut microbiota (GM) is a complex microecosystem containing various microorganisms^1^ that participate in diverse biological processes in hosts, such as nutrient metabolism,^2,3^ immune development,^4,5^ and neurological regulation.^6,7^ Using high-throughput sequencing, an increasing number of studies report the GM characteristics in humans that underlie different health statuses – such as type II diabetes,^8^ Crohn’s disease,^9^ and Alzheimer’s disease (AD)^10^ – as well as the associations between the GM components and physiological indices of hosts.^11^ Notably, the intimate association between the GM and brain has received extensive attention, prompting reports of the “gut–brain axis” in studies of various neurological diseases such as AD,^12^ Parkinson’s disease,^13^ depression,^14^ etc.^15^ Previous studies identified that the GM is involved in the pathogeneses of central nervous system diseases via the secretion of short-chain fatty acids (e.g., acetate, propionate, and butyric acid)^16–18^ and neuroinflammatory modulators (e.g., dopamine, serotonin, and GABA).^12,18^ However, GM interactive networks, which are important for the maintenance and intervention of the microecosystem, are seldom reported.

GM interactive network has the advantage of characterizing complicated microecological relationships including cooperation, mutualism, competition, antagonism, etc.; meanwhile, the nodes and edges in a network can represent the bacteria and relationships in a microecosystem, respectively.^19,20^ Thus, utilizing network theory would assist explorations of GM interactive networks aiming to decipher the complicated interactions among gut bacteria and detect the hub bacteria of a microecosystem.^19^ Accordingly, we can infer the stability of an ecosystem and evaluate the impacts of environmental factors on community dynamics based on the structural features of an interactive network, such as species richness, connectivity, interaction strengths, etc.^21^ In addition, bacterial functional analysis combined with metabolic analysis would enable further investigation of the specific roles of hub bacteria in the pathogeneses of various diseases, providing insights into the mechanism of gut–brain communication.^6^

Complex network analysis is a powerful tool for detecting the ecological interactions of a community^22^ and aids investigations of the topological and structural features of GM networks. This type of analysis is widely applied in macroecological research (e.g., studies on food chain, animal, and plant networks) and has advanced our understanding of the alteration and co-evolution of various species.^23–25^ Complex network analysis was recently applied to the study of microecosystems including microbial co-occurrence networks in marine,^26^ water,^27^ soil,^28^ and human gut environments. One recent study applied this approach to soil microbial communities to evaluate the complexity and stability of soil microbial networks associated with climate change.^29^ A microbial co-occurrence network is often constructed to detect gut bacterial relationships under different health states such as cholestasis^30^ and inflammatory bowel disease.^31^ Some studies also determined the topological properties of the GM co-occurrence network in AD model mice at eight months old and report that the GM network in these mice has fewer edges, decreased correlation density, and lower transitivity compared to that in control mice.^32^ However, the dynamic changes of GM complex networks and their biological applications in disease intervention have yet to be explored. In addition, newly developed algorithms have yielded a comprehensive understanding of network structures.^22,33,34^ For example, several frameworks can detect the fragility, stability, and controllability of complex networks and identify the time-dependent driver nodes for system intervention.^22,33,34^ Nevertheless, the various existing algorithms and tools for complex network analysis are seldom used to explore GM co-occurrence networks during the progression of various neurological disorders. In addition, it is unknown whether the window period and target bacteria for disease intervention can be inferred through GM networks.

Accordingly, in this study, we hypothesized the following: (a) that the GM networks differ between AD and wild-type (WT) mice, (b) the topological features of GM networks change during AD development, and (c) complex network-based GM modeling can be used to explore the optimal window period and target bacteria for GM interventions during AD progression.

## Results

### Dynamic changes of the gut microbiota in mice

**Figure 1.**
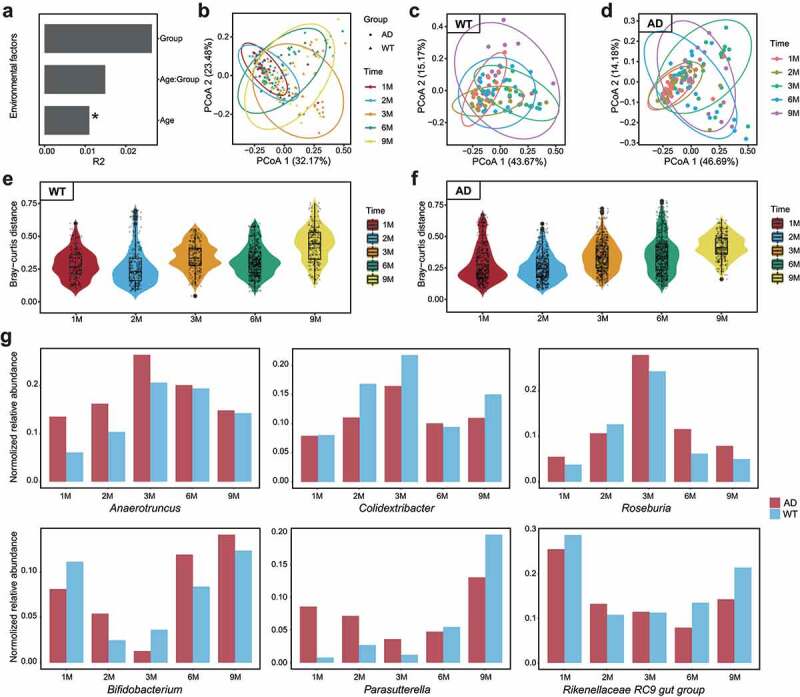
Longitudinal changes of gut microbiota components in Alzheimer’s disease and wild-type mice during development. (a). Impacts of environmental factors on gut microbiota (GM) differences observed by permutational multivariate analysis of variance (PERMANOVA) analysis. The contributions of environmental factors to GM variance were quantified with R2, and age significantly influenced GM differences. (b). Results of Bray–Curtis distance-based principal coordinates analysis (PCoA) with all samples; dots and triangles indicate samples from the Alzheimer’s disease (AD) and wild-type (WT) mice, respectively. Colors indicate mouse age: red, one month; blue, two months; Orange, three months; green, six months; yellow, nine months. Circles contain samples with 95% confidence intervals for the groups stratified by age. (c, d) PCoA results of the WT (c) and AD (d) samples. Colors indicate mouse age: pink, one month; grass green, two months; green, three months; blue, six months; purple, nine months. (e, f) Inter-group GM differences of the WT (e) and AD (f) mice. Horizontal and vertical axes indicate mouse age and the Bray–Curtis distance, respectively. (g) Fluctuation patterns of bacterial genera in the AD and WT mice. The min–max-normalized genera abundances in the AD and WT mice are indicated by red and blue, respectively.

This study explored the dynamic changes of the GM in AD and WT mice at one, two, three, six, and nine months of age. Permutational multivariate analysis of variance (PERMANOVA) analysis detected the impacts of environmental factors on GM composition, indicating that age has the strongest influence on GM components (*P* < .05, Figure 1a). Bray–Curtis distance-based principal coordinates analysis (PCoA) yielded consistent results – specifically, the GM from AD and WT mice of the same age clustered together (Figure 1b). To examine the impacts of age on GM components, we further performed PCoA analyses separately on the AD and WT mice. The GM from the WT mice showed obvious changes with increasing age (Figure 1c). In contrast, the GM from AD mice changed only slightly within the first two months after birth, then changed remarkably from the third to ninth month of age (Figure 1d). In addition, examination of intergroup GM differences revealed that individual GM diversity increased with age in both AD and WT mice (Figure 1e,f). These findings suggest that mice with neurological dysfunctions have GM developmental patterns distinct from those of WT mice.

We then selected the top 20 genera from the mice and examined their changes over time (Figure 1g and Supplementary Figure S1). In mouse gut, the dominant bacterial taxa were *Bifidobacterium, Lactobacillus, Mucispirillum, Muribaculum*, the *Lachnospiraceae* NK4A136 group, and the *Rikenellaceae* RC9 gut group, which accounted for 69.07 ± 15.76% (mean ± SD) of the GM (Supplementary Figure S2). Among the GM components, some bacteria exhibited different fluctuation patterns between the AD and WT mice. We summarized these differences into four categories (Figure 1g): (1) bacteria with consistently higher abundance in the AD mice (e.g., *Anaerotruncus* and *Roseburia*); (2) bacteria with consistently lower abundance in the AD mice (e.g., *Colidextribacter*); (3) bacteria whose abundance exhibited a delayed time inflection point in the AD mice (e.g., *Bifidobacterium* and the *Rikenellaceae* RC9 gut group); and (4) bacteria with disordered fluctuation patterns (e.g., *Parasutterella*). These findings reveal the temporal fluctuation patterns of GM in the AD and WT mice, indicating developmental differences in the GM during neurodegenerative disease progression.

### Topological features and hub bacteria of the gut microbiota co-occurrence networks

**Figure 2.**
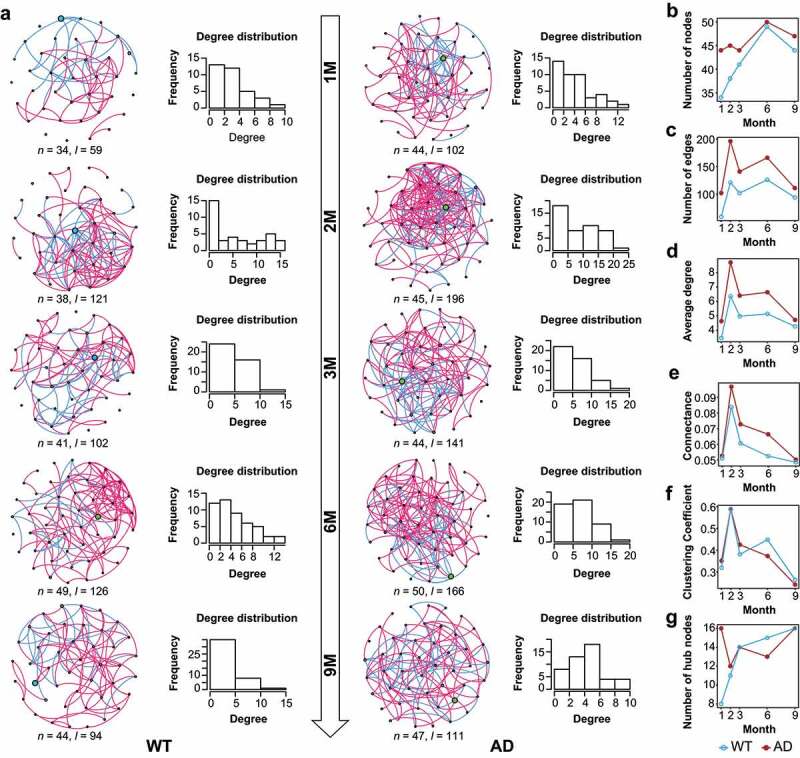
Dynamic changes of the gut microbiota co-occurrence networks in Alzheimer’s disease and wild-type mice. (a) Gut microbiota (GM) co-occurrence networks with respect to mouse age in the Alzheimer’s disease (AD) and wild-type (WT) mice. In the networks, each node indicates a genus, and its size indicates relative abundance. Pink and green edges indicate positive and negative correlations, respectively. Histograms show the degree distributions of nodes of the networks. (b–g) Topological features of GM co-occurrence networks in the mice, including the dynamic changes of nodes (b), edges (c), average degree (d), clustering coefficient (e), connectance (f), and hub nodes (g). The WT and AD groups are indicated by blue and red, respectively.

Given that microbial communities involve complex synergistic and antagonistic relationships,^35^ we constructed GM co-occurrence networks for the AD and WT mice at different time points and applied the complex network approach for our topological and hub bacteria analyses of the GM networks. We considered the genera and their correlations as nodes and links in the GM networks. Based on the degree distributions of the nodes, we discovered that the distribution histogram of the networks fits the power-law model in the WT mice, suggesting scale-free features (Figure 2a). Meanwhile, in the AD mice, the distributions of the nodes’ degrees conformed to the power-law model in the first three months of age but conformed to the Poisson model at six and nine months. These results suggest that the GM networks changed from scale-free networks to random graphs in the AD mice during development (Figure 2a). As neurodegenerative behaviors in AD mice normally appear after six months of age,^36^ these results indicate that the alteration of GM topological features occurred before the appearance of clinical phenotypes, providing us with a potential tool for AD prediction. In addition, compared to the WT mice, the AD mice had larger GM networks including more nodes and links (Figure 2b,c). Accordingly, the networks of the AD mice were more complex – for example, having a higher average degree and connectance (Figure 2d,e). The larger GM network of the AD mice is likely related to their higher bacterial diversity. However, the clustering coefficients of the networks in the AD mice decreased at six and nine months (Figure 2f) and were accompanied by fewer hub bacteria at six months (Figure 2g). The fluctuating rhythm of clustering coefficients is consistent with the alteration of the network topological features, hinting at the sparse GM network and loss of influence of hub bacteria at the end stage of AD progression. Therefore, the potential window period for GM intervention in AD mice is likely in the first three months. Although the longitudinal tracing of the GM networks helped reveal the critical stage of GM alteration in AD mice, this broad window period must be narrowed for GM-targeting interventions.

We further explored the hub bacteria for each GM co-occurrence network based on the node degrees. Notably, *Colidextribacter* and *Roseburia* were the hub taxa that maintained the GM networks across different time points in the WT and AD mice, respectively (Figure 2a). Besides these two taxa, other hub taxa in the networks changed over time and differed between the AD and WT mice. In the GM networks of the AD mice, the specific hub taxon at one and two months was the *Rikenellaceae* RC9 gut group, that at three and six months was *Lactobacillus*, and that at nine months was *Akkermansia* (Figure 2a). These findings provide the hub bacteria of the GM network as potential candidates for GM manipulation, assisting interventions for neurological disorders via the gut–brain axis.

### Assessment of the stability, vulnerability, and computational simulation-based robustness of the gut microbiota networks

**Figure 3.**
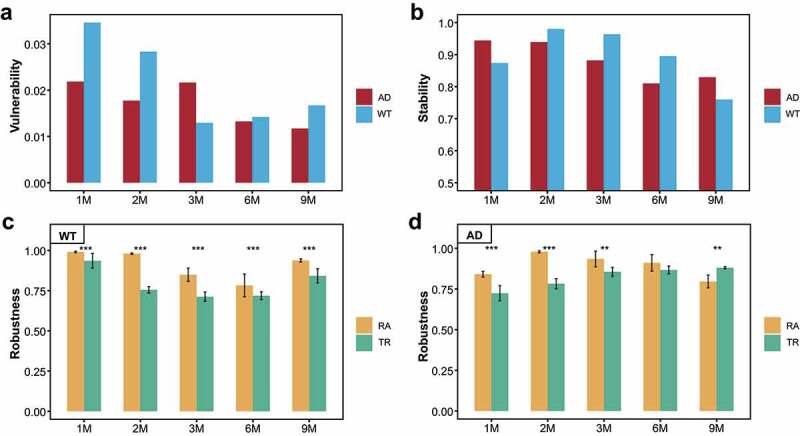
Vulnerability, stability, and robustness assessment of the gut microbiota co-occurrence networks. (a, b) Dynamic changes of the vulnerability (a) and stability (b) of the gut microbiota (GM) co-occurrence networks with respect to mouse age. Red and blue indicate the Alzheimer’s disease (AD) and wild-type (WT) mice, respectively. (c, d) Computational simulation-based robustness assessment results of the GM co-occurrence networks in the WT (c) and AD (d) mice. Different strategies are indicated by different colors. TR: hub bacteria-based target removal; RA: non-hub bacteria-based random attack. ** *P* < .01, *** *P* < .001.

**Figure 4.**
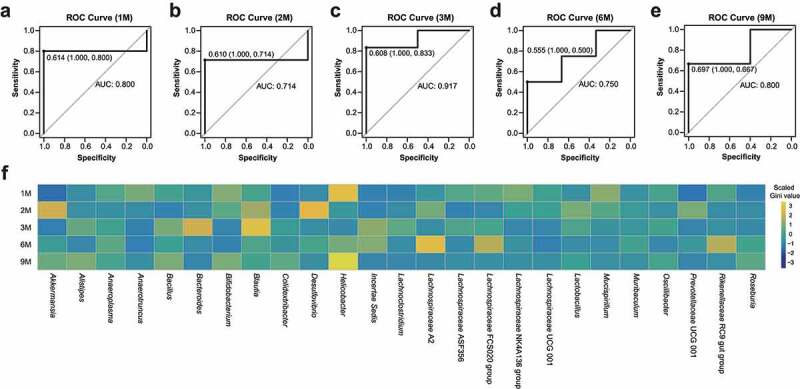
Random-forest models to distinguish the gut microbiota between Alzheimer’s disease and wild-type mice during development. (a–e) Receiver operating characteristic (ROC) curves and areas under curve (AUCs) for the random-forest models with three-fold cross-validation at the one, two, three, six, and nine months of age. (f) Heatmap of the scaled Gini values in different random-forest models with respect to mouse age. Yellow and blue panels indicate bacteria with high and low Gini values, respectively.

We subsequently assessed the stability and vulnerability of the GM networks to determine the optimal window period for GM intervention using the abundance-weighted mean interaction strength algorithm (detailed in the *Methods*). We first evaluated the vulnerability of the GM networks in the mice (Figure 3a). The results show that the GM networks of the WT and AD mice were most vulnerable at one and three months of age, respectively (Figure 3a). Next, we evaluated the stability of the GM networks at different ages in the AD and WT mice (Figure 3b). Along with the GM fluctuations, the stability of the GM networks peaked at two months in WT mice, gradually decreasing thereafter (Figure 3b). Intriguingly, the stability of the GM network peaked at one month in the AD mice and reached a nadir at six months. Moreover, compared to the WT mice, the GM network stability in the AD mice was lower at two, three, and six months of age but higher at one and nine months of age (Figure 3b). The stability and vulnerability assessment results collectively suggest that three months of age is the optimal window period for GM intervention in AD mice. To further examine the findings of the optimal window period, we constructed random-forest models to distinguish the GM between the AD and WT mice from a machine learning perspective (Figure 4). At different ages, the areas under the curve (AUC) for the random-forest models peaked at three months of age (AUC = 0.917) (Figure 4a–e), which is consistent with the findings of the complex network-based assessment of the GM intervention window.

Furthermore, we used the hub bacteria-based target removal (TR) method and non-hub bacteria-based random attack (RA) method to evaluate the impacts of the hub bacteria on the robustness of the GM networks. Repeating the simulations 10 times revealed that the TR method significantly decreased GM network robustness compared to the RA method in the WT mice (*P* < .05, Figure 3c). Notably, in the AD mice, the robustness of the GM networks diminished significantly under the TR method in the first three months of age, whereas the RA method reduced the robustness of the GM network at nine months (Figure 3d). As the GM networks in the AD mice changed from scale-free networks to random graphs from six to nine months old, this indicates that the TR method is the optimal method for intervention in the scale-free GM network. Based on the computational simulations, we also deduced that the hub bacterial taxa in the third month (i.e., *Muribaculum, Lachnospiraceae* UCG-001, *Oscillibacter, Desulfovibrio*, etc.) were the optimal targets for GM network manipulation. Thus, this computational simulation approach was convenient for determining the optimal stage and target bacteria for GM intervention.

### Neurological impacts of the hub bacteria on the hosts

**Figure 5.**
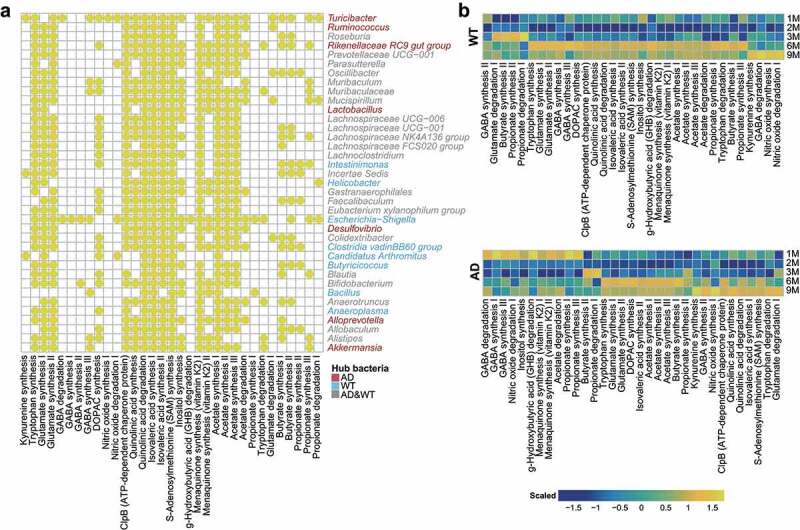
Distribution of gut–brain modules in Alzheimer’s disease and wild-type mice. (a) Presence of gut–brain modules (GBMs) among the hub bacteria of the Alzheimer’s disease (AD) and wild-type (WT) mice. Horizontal and vertical axes show the GBMs and genera, respectively. Yellow circles indicate the presence of GBMs in the genus. Genera marked in red, blue, and gray are the hub bacteria present in the AD mice, WT mice, and both, respectively. (b) Fluctuations of GBMs with respect to age in the AD and WT mice. The GBMs were sorted according to their peak abundances at different ages in the AD and WT mice. Orange and blue indicate high and low abundance, respectively.

**Figure 6.**
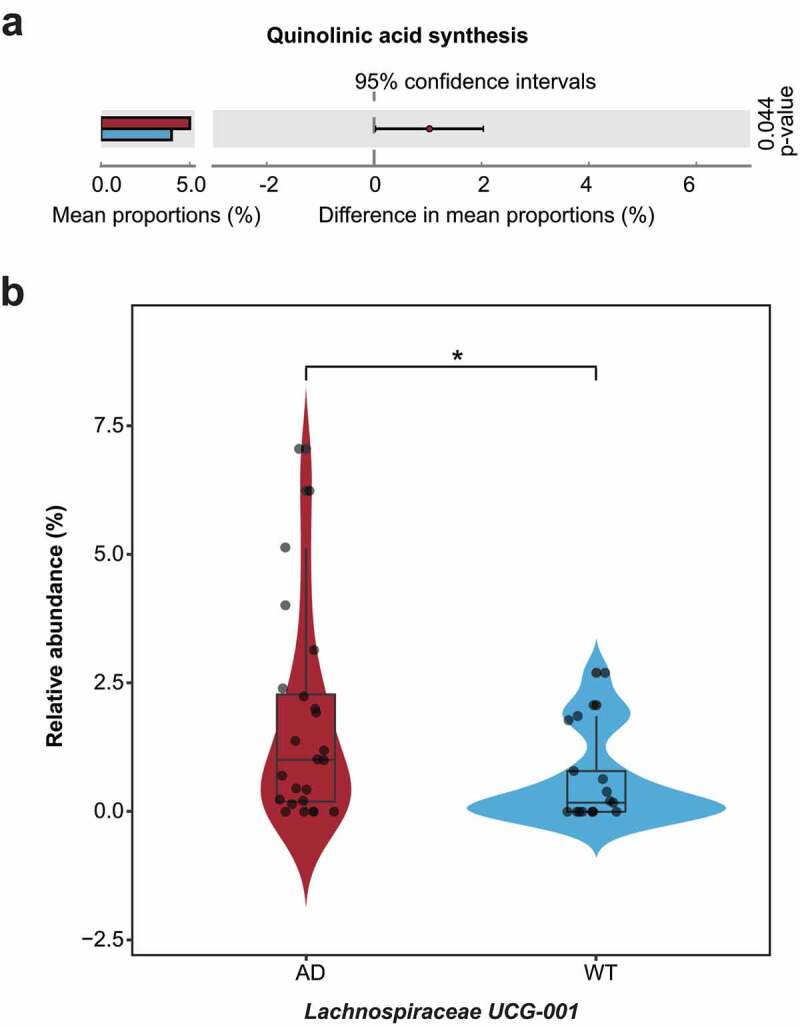
Differentially enriched neuroactive functions and corresponding hub bacteria in three-month-old Alzheimer’s disease mice. (a) Differentially enriched gut–brain modules (GBMs) in the Alzheimer’s disease (AD) mice at three months of age. The mean proportion of the GBM (left) and 95% confidence intervals in the enriched group (right) are shown. The AD and wild-type (WT) groups are indicated by red and blue, respectively. (b) Comparison of the hub bacteria responsible for the differentially enriched GBMs between the AD and WT mice. The AD and WT mice are indicated by red and blue, respectively. * *P* < .05.

By using PICRUSt software^37^ and the gut–brain modules (GBMs) database,^38^ we aimed to illustrate the functional distributions of the GM, detect the neurological impacts of the hub bacteria on the hosts, and explore their dynamic temporal changes (Figure 5 and Supplementary Figure S3). *Turicibacter* – the hub bacterial taxa in the AD mice at one, six, and nine months old – is involved in kynurenine synthesis, GABA degradation, and quinolinic acid synthesis, suggesting that it plays essential roles in the nervous system and AD pathogenesis (Figure 5a). In contrast, *Butyricicoccus* – the hub bacterial taxa in the WT mice at six months old – participates in butyrate synthesis and tryptophan synthesis, which can suppress neuroinflammation in hosts (Figure 5a). After analyzing the dynamic changes of these neural-related GM functions, we observed “peak shifts” of some functions in the AD mice compared with the WT mice (Figure 5b). For example, the abundance peaks of GABA synthesis, menaquinone synthesis, propionate synthesis, acetate degradation, and hydroxybutyrate degradation shifted from six months of age in the WT mice to one month of age in the AD mice (Figure 5b). In contrast, the abundance peaks of quinolinic acid degradation, isovaleric acid synthesis, and S-adenosylmethionine synthesis shifted from six months of age in the WT mice to nine months of age in the AD mice (Figure 5b). Moreover, these three GBMs existed in a majority of the hub bacteria from the AD and WT mice (Supplementary Figure S4). Previous studies demonstrate that quinolinic and isovaleric acid play important roles in AD pathogenesis and synaptic neurotransmitter release, respectively.^39,40^ Therefore, the GM function alterations caused by GM fluctuations in the AD mice suggest that they are associated with the altered host neural responses. In addition, we compared the abundance of the GBMs between the AD and WT mice at three months old and detected the hub bacteria that correspond to the differentially enriched GBMs (Figure 6). Using STAMP software,^41^ we discovered that the function of quinolinic acid synthesis was significantly enriched in the AD mice (Figure 6a) and that its corresponding bacterial taxa (i.e., *Lachnospiraceae* UCG-001) was also significantly enriched in the GM (Figure 6b). These results suggest that *Lachnospiraceae* UCG-001 may be an important target for GM intervention in three-month-old AD mice. Furthermore, integrating functional and network analyses may help narrow the scope of GM-targeting interventions.

## Discussion

**Figure 7.**
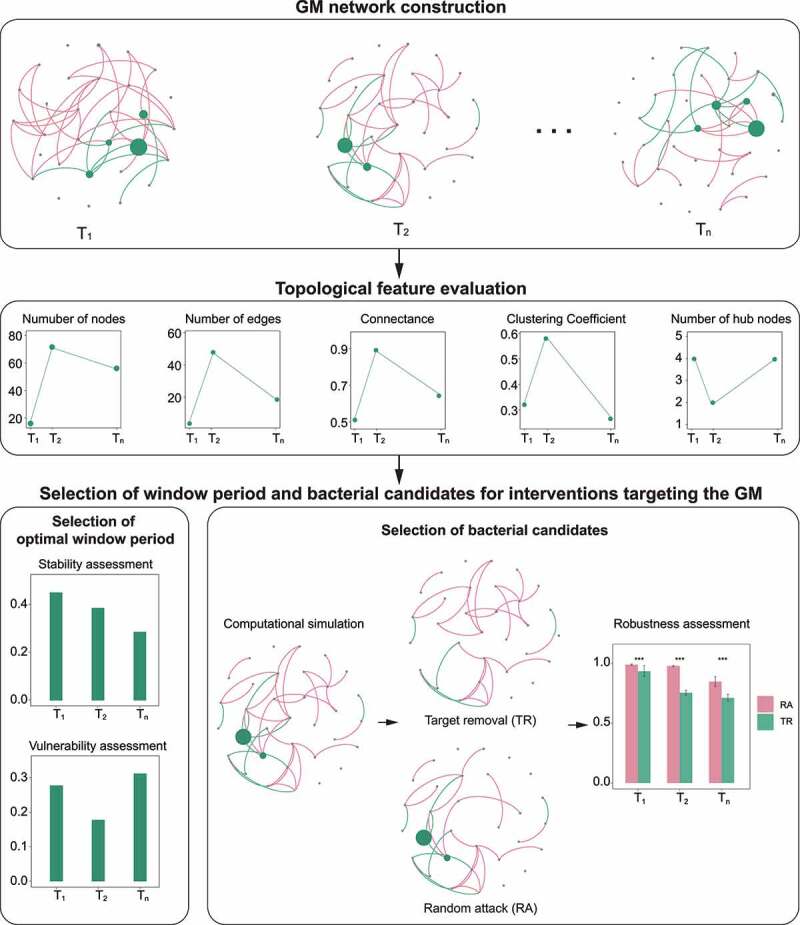
Framework for gut microbiota network modeling. The framework primarily comprises four components: GM network construction, topological feature evaluation, and the selection of window period and bacterial candidates for interventions targeting the GM.

Diverse microorganisms in the microecosystem form complex microbial networks and affect their hosts’ health status through exchanges of nutrition, energy, and information.^22^ Previous studies have mainly focused on the alterations of GM components and their associations with their hosts^1,17,42^ but seldom report the features of microbial interactive networks or the optimal time window and bacterial taxa for GM-targeting interventions. Here, we constructed a complex network-based GM modeling framework to explore the topological features of GM networks in AD and WT mice and detected the dynamic changes of these networks and their hub bacteria during mouse development (Figure 7). In addition, we performed computational simulations to evaluate the impacts of the hub bacteria on the robustness of the GM networks and determine the optimal intervention window and candidate target bacteria for GM-targeting interventions in the AD mice, providing essential references for disease intervention from the perspective of the GM.

Selecting the optimal intervention window is a challenging but critical decision in clinical medicine. However, an adequate theoretical framework to predict this optimal intervention period is still lacking. Therefore, this study aimed to construct a complex network-based GM modeling strategy in order to aid the development of novel treatments for GM-related disorders. We comprehensively assessed the topological features, stability, and vulnerability of the GM co-occurrence networks in mice to determine the GM intervention window, which yielded three noteworthy findings. First, the GM networks in the AD mice were scale-free networks in the first three months after birth, indicating that these networks can be manipulated through their hub bacteria.^43^ Second, the vulnerability of the GM network peaked at three months of age in the AD mice, narrowing the intervention window. Third, the stability of the GM network in the AD mice in the third month was lower than that in the first and second months. These results collectively indicate that in the AD mice, the third month after birth is the optimal window period for GM-targeting interventions. In addition, we constructed supervised machine learning models with bacterial profiling, which revealed that the AUCs of the random-forest model peaked in the third month of mouse development. The results are concordant with those of the GM modeling analysis, further supporting three months of age as the optimal intervention window. Therefore, the assessment of the topological features, stability, and vulnerability of GM networks is a powerful tool for detecting the optimal window period for GM-targeting interventions and understanding the inner interactions of the GM.

We subsequently applied computational simulation methods to explore the possibility of the hub bacteria as targets for GM manipulation and disease intervention. We simulated the removal of the hub or non-hub bacteria from the GM co-occurrence networks, detected changes in the robustness of the networks, and identified the target bacterial taxa for GM-targeting interventions. The simulations of the TR and RA methods revealed that the former could effectively reduce the robustness of scale-free networks. In contrast, for random networks, the TR method had no advantage in reducing network robustness compared to the RA method. Based on these observations, we deduced that the interventions targeting the hub bacteria in the AD mice at the optimal window period (i.e., three months old) – *Muribaculum, Lachnospiraceae* UCG-001, *Oscillibacter*, etc. – would significantly alter the structure of the GM network. Meanwhile, from a biological perspective, the hub bacteria identified in our analysis are reported to have important functions in modulating the nervous system in their hosts.^44–46^ For example, the abundance of *Oscillibacter* is positively associated with depressive behaviors in humans, probably owing to the binding of GABA receptor and modulation of the GABA system by *Oscillibacter*-derived valeric acid.^44,46,47^ In addition, *Lachnospiraceae*, a dominant taxon in the human GM, has roles in short chain fatty acid production and anti-inflammation,^45,48^ and is negatively associated with amyloid and phospho-tau levels in AD pathology.^49^ Another hub bacterial taxa, *Muribaculum*, is enriched in AD mice and negatively associated with cognitive decline in an AD mouse model.^50,51^ Nevertheless, the underlying mechanisms by which these hub bacteria regulate brain functions are not well understood. Thus, *in silico* guided biological characterization of hub bacteria would be an intriguing direction for future research.

After uncovering the bacterial candidates involved in the GM alteration in mice, we further identified the responsible bacterium by combining the computational simulation with GM functional analysis. We found that *Lachnospiraceae* UCG-001, which was enriched in the AD mice, was the potential keystone bacterial taxa owing to its contributions to quinolinic acid synthesis, which is an enriched function in the AD mice. Previous studies demonstrate that quinolinic acid is involved in AD pathogenesis via excitotoxic effects, metabolic damage, inflammatory responses, and neuronal and astrocytic apoptosis.^39,52^ Hence, the computational simulation approach developed in this study can help identify target bacteria and provides a theoretical basis for disease intervention from the perspective of GM network manipulation.

Besides the characteristics of the GM networks, our study also revealed the temporal compositional and functional changes of the GM and that age is the most important environmental factor for GM variation. As the AD and WT mice in this study were reared in the same environment, the AD-related genotype likely affected the GM components, thereby influencing the permeability of the intestinal mucosa and brain functioning via mutual gut–brain communications.^12,53^ Although this study revealed distinct hub bacteria in WT and AD mice at different ages, their effects on disease progression require further investigation. As a persistent hub bacterial taxon in the WT mice, *Colidextribacter* can reduce the risk of depression by repressing neuroinflammation in hosts.^54^ On the other hand, Zheng et al. report that the *Rikenellaceae* RC9 gut group, a hub bacterial taxon in the AD mice, is enriched in schizophrenia model mice, suggesting their impact on neuromodulation.^55^ These findings suggest that changes in the hub bacteria could both adjust the GM networks and affect host health status.

Thus, the above results indicate that GM-based approaches for disease intervention are a promising and challenging research direction. Previous reports indicate that prebiotics, specific probiotics, and dietary habits could be effective strategies to modify the GM components.^56^ Specific hub bacteria can be targeted *in vivo* by ecological antagonism or phage therapy.^57,58^ Through nutrient competition, bacteriocin secretion, and toxic metabolites, some bacteria could prevent the overgrowth of specific pathogens.^58^ For example, microcins produced by *Escherichia coli* could repress some *Enterobacteriaceae* species.^59^ On the other hand, bacteriophages also have potential to eliminate specific bacteria.^57^ In a recent study, Eran Elinav et al. demonstrated the inhibitory effect of phage consortia on inflammatory bowel disease-associated bacteria.^60^ However, several challenges (e.g., dosage, safety, and efficacy) must be overcome prior to the clinical application of bacteria-manipulating therapies.^57,58^ Nevertheless, the comprehensive analysis approach established in this longitudinal study might reveal the key bacteria that affect GM homeostasis during disease progression, providing deeper insights into the bidirectional GM–host interactions in disease pathogenesis.

Regarding the relationship between disease severity and breeding condition, compared to APP/PS1 mice (a mouse model of AD) in a normal breeding environment, APP/PS1 mice bred in germ-free conditions or treated with antibiotics exhibit attenuated AD-related pathological phenotypes (e.g., compromised amyloid deposition, neuroinflammation, and cognitive decline).^61–63^ In addition, our previous study demonstrates that GM alterations precede the development of key pathological features of AD.^64^ Together, these findings strongly support the notion that GM alterations in AD mice initially caused by heredity in turn exacerbate disease progression.

The benefits of mouse models include well-controlled breeding environments and the convenience of longitudinal tracing of GM changes. Therefore, we utilized data from mice to generate a complex network-based GM modeling strategy, which is theoretically sound and rigorous, to improve our understanding of the competitive and cooperative relationships of microecosystems. Accordingly, such modeling in mice provides valuable experience for future explorations of optimal window periods for GM-targeting interventions in humans. By tracing the dynamic GM changes during disease progression, this strategy determined the optimal window period for GM-targeting interventions, which is essential for the effectiveness of clinical interventions. In addition, combining computational simulations with bacterial functional analysis can aid the discovery of target bacteria for GM-targeting interventions, leading to opportunities for future investigations of the pathogeneses of various diseases.

To adjust for the individual age-related GM diversity in mice and validate our current findings in future studies, we will utilize a larger sample size. Moreover, *in vivo* experiments based on the results of computational analysis may help us assess the feasibility of our strategy for clinical applications. The GM functions and metabolites (e.g., quinolinic and isovaleric acid) that differ between the AD and WT mice are powerful indicators of the roles of the GM in AD pathogenesis.^39,40^ Nevertheless, these GM metabolic changes must be verified by mass spectrometry, and their functions need to be explored through *in vivo* experiments. Finally, findings about the GM in mice are not fully representative of the GM in humans because of differences in the genetic backgrounds, physiological features of gastrointestinal tracts, and dietary habits between mice and humans.^65^ Compared to humans, the intestinal tract in mice has lower pH, lower oxygen tension levels, and different glycan profiles, which are partially responsible for the differential GM compositions between species.^65^ Furthermore, intestinal transit time (the time food takes to travel through the digestive tract) is shorter in mice than in humans, which also contributes to GM differences.^65^ Therefore, the application of the GM network modeling strategy reported herein must be validated and optimized for patients with AD or other neurodegenerative diseases to detect specific intervention windows and target bacteria.

In summary, we constructed a complex network-based modeling approach to investigate the GM, explore dynamic GM network changes in AD and WT mice, and identify the optimal window period and bacterial candidates for GM-targeting interventions in the AD mice. Theoretically, our study integrates complex network theory with longitudinal GM research, opening new avenues for manipulating the microbial community in hosts. Practically, we can extend the network modeling approach to neurological diseases or even broader clinical research fields, which would facilitate the faster and more efficient development of disease interventions.

## Methods

### Data acquisition

This study used previously published 16S rRNA amplicon data obtained from APP/PS1 mice (i.e., B6C3-Tg [APPswe, PSEN1dE9] 85Dbo/J) and their WT littermates.^64^ In brief, we collected 204 stool samples from the AD and WT mice at one month (*n* = 21 and 15, respectively), two months (*n* = 23 and 17, respectively), three months (*n* = 24 and 17, respectively), six months (*n* = 26 and 25, respectively), and nine months (*n* = 18 and 18, respectively). We then isolated microbial DNA from the stool samples (DNeasy PowerSoil Kit, QIAGEN, Germantown, MD, USA), amplified the 16S rRNA V4 region (AP221-02, TransGen Biotech, Beijing, China), and performed 200-nt paired-end sequencing with the HiSeq 1500 platform (Illumina, San Diego, CA, USA). This study was performed in accordance with the recommendations of the National Care and Use of Animals Guidelines (China) and approved by the Institutional Animal Care and Use Committee (IACUC) of the Shenzhen Institute of Advanced Technology, Chinese Academy of Sciences.

### Data filtration and taxonomical annotation

We used an in-house script to filter out the low-quality reads from the raw sequencing data when they contained >10 low-quality bases (<Q30) or 15 adapter bases. We then made taxonomical annotations with the clean reads using QIIME2 software (version 2021.11.0).^66,67^ First, we merged the paired-end reads into tags based on their overlaps using vsearch software (version 2.7.0).^68^ Second, we obtained the amplicon sequence variants after tag clustering with deblur software (version 1.1.0).^69^ Third, we used the sklearn-based taxonomy classifier and trained SILVA Database (version 138.99) to perform taxonomical annotation of the amplicon sequence variants.^70^ Last, we profiled the samples according to their taxonomical annotation results.

### Functional prediction and gut–brain module analysis

We obtained the distributions of GM functions with the amplicon sequence variants using PICRUSt2 software (version 2.3.0).^37^ The functional profile contained the abundances of KEGG Orthology and metabolic pathways for all samples. Using the KEGG Orthology profile, we further detected the neurological-related functions in the GM through the previously published GBMs database.^38^ First, we collected the KEGG Orthology list for each GBM and calculated the abundance of GBMs for each sample through the KEGG Orthology abundance. To obtain the relationships between the GBMs and the bacterial taxa, we then bridged the taxa and the GBMs through the amplicon sequence variants.

### Construction of random-forest models

We constructed the AD-risk models using the “randomForest” package in R and the GM profiles at one, two, three, six, and nine months of age. For each model, we extracted the GM profiles from the AD and WT mice at the same age and randomly divided them into three groups: two groups, which comprised the training group, and one test group. We then detected the optimal variance and tree numbers using the genus profiling from the training group and constructed the AD-risk model using the “randomForest” function. We also assessed the Gini value for each genus in the risk model. Finally, we detected the accuracy and sensitivity of the model using the “predict” function and presented the results as receiver operating characteristic (ROC) curves with AUCs.

### Gut microbiota co-occurrence network construction and characterization

We constructed the GM co-occurrence networks for the AD and WT mice at one, two, three, six, and nine months of age. With the GM profiles for each group, we calculated the Spearman correlation coefficients between genera using the “psych” package in R and kept the correlations *r* < −0.4 or *r* > 0.4 (*P* < .05). We then plotted the GM co-occurrence networks using Gephi (version 0.9.2).^71^ To characterize the topological features of the GM co-occurrence networks, we used the “igraph” package in R and analyzed the numbers of nodes and edges, average degree, degree distribution, clustering coefficient, and connectance in each network. In the GM co-occurrence networks, each node indicated a genus, and each link indicated a relationship between genera. Furthermore, we defined the bacteria as hub nodes when their degrees exceeded the third quartile of the degree in a network.

### Network stability evaluation

RTIS_A_2168546Before evaluating the stability of the GM networks, we first detected the effect of the nodes in a network by using the abundance-weighted mean interaction strength ($wMIS_{i}$) index.^29^ We calculated the $wMIS_{i}$ index for each node in a network with the following formula:

$$wMIS_{i}=\frac{\sum_{j\neq i}b_{j}\left|R_{i,j}\right|}{\sum_{j\neq i}b_{j}}$$

where *i* is a node in a network, *j* is the node connected to node *i, b_j_* is the relative abundance of node *j*, and *R_i,j_* is the Spearman correlation coefficient between nodes *i* and *j*. To evaluate the stability of the networks throughout mouse development, we first detected the core nodes in the networks, wherein the core nodes were defined as the consistent bacteria existing in the GM networks of the AD or WT mice across different time points. We then calculated the stability of the networks ($S_{a}$) according to the $wMIS_{i}$ index using the following formula:

$$S_{a}=\frac{\sum_{i=1}^{m}wMIS_{i}}{\sum_{j=1}^{n}wMIS_{j}}$$

where *m* is the core nodes in network *a* and *n* is all nodes in network *a*.

### Network vulnerability evaluation

We also evaluated the vulnerability of the GM networks by calculating the maximal global efficacy decreasing ratio (mEDR). Before determining the mEDR, we first calculated the averaged efficacy of a network ($E_{a}$), which implies the transferring speed of information in the network. We calculated *E_a_* using the following formula:

$$E_{a}=\frac{1}{n\left(n-1\right)}\underset{i\neq j}{\sum }\frac{1}{d_{i,j}}$$

where *n* is the number of nodes in network *a* and $d_{i,j}$ is the number of edges in the shortest path between nodes *i* and *j*. We then removed each node from the network one by one, evaluated the altered ${E_{a}}^{\prime }$ after each node’s removal, and selected the maximal EDR as the mEDR using the following formula:

$$mEDR=max\left(\frac{E_{a}-{E_{a}}^{\prime }}{E_{a}}\right)$$

### Computational simulation and network robustness evaluation

We simulated the processes of hub bacteria-based TR and non-hub bacteria-based RA in the GM co-occurrence network and defined the network robustness ($R_{a}$) as the ratio of remaining bacterial $wMIS_{i}$ to the total $wMIS_{i}$ of a network after computational simulation. We calculated $R_{a}$ using the following formula:

$$R_{a}=\frac{\;\sum_{j=1}^{n}wMIS_{j}-\sum_{i=1}^{m}wMIS_{i}}{\sum_{j=1}^{n}wMIS_{j}}$$

where *m* is the removed nodes in the network *a* and *n* is all nodes in network *a*. For the TR simulation, we randomly removed half of the hub bacteria from the network and calculated the ${R_{a}}^{\prime }$ of the network. We also randomly removed the same number of non-hub bacteria from the network and calculated the $R_{a}^{{\;}^{\prime \prime }}$ of the network. Upon repeating this process 10 times, we compared the network robustness between the TR and RA methods.

### Statistical analysis

We evaluated the data size with rarefaction curves using the “vegan” package in R (bootstrap = 500, Supplementary Figure S5). We then calculated the Bray–Curtis distances among the samples (also using the “vegan” package in R) and applied the Wilcoxon rank-sum test to examine the differences between groups. We assessed the impacts of environmental factors on GM composition by PERMANOVA analysis with 9,999 permutations. Based on the Bray–Curtis distances, we performed PCoA. We adopted the min–max normalization method to analyze bacterial abundance when detecting the fluctuation pattern of the bacteria. To identify differentially enriched GBMs between groups, we used STAMP software (version 2.1.3)^41^ with a two-tailed Welch’s *t*-test (*P* < .05). We sorted the GBMs in the AD and WT mice according to their scaled relative abundance across different time points and visualized the results as heatmaps created using the “pheatmap” package in R. We adjusted the statistical results from the Wilcoxon rank-sum test and Spearman correlation analysis according to the Benjamini and Hochberg method (false discovery rate [FDR] < 0.05) using the “p.adjust” package in R. We plotted the ROC curves using the “pROC” package in R and generated other corresponding figures using the “ggplot2” package in R.

## Supplementary Material

Supplemental Material

## Data Availability

The complex network-based GM modeling algorithm codes and computational simulation process are publicly available in the GitHub repository (https://github.com/liyinhu/GM_network_modeling). The 16S rRNA sequencing data have been deposited in the NCBI Sequence Read Archive (SRA) repository under BioProject accession number PRJNA543965 (https://www.ncbi.nlm.nih.gov/bioproject/543965). All other data are available from the authors upon reasonable request.
