# Supplementary figures and images for: Dynamic network modeling of gut microbiota during Alzheimer’s disease progression in mice
Source: Gut Microbes. 2023 Feb 1;15(1):2172672. doi: 10.1080/19490976.2023.2172672 (PMC9897752; doi:10.1080/19490976.2023.2172672)

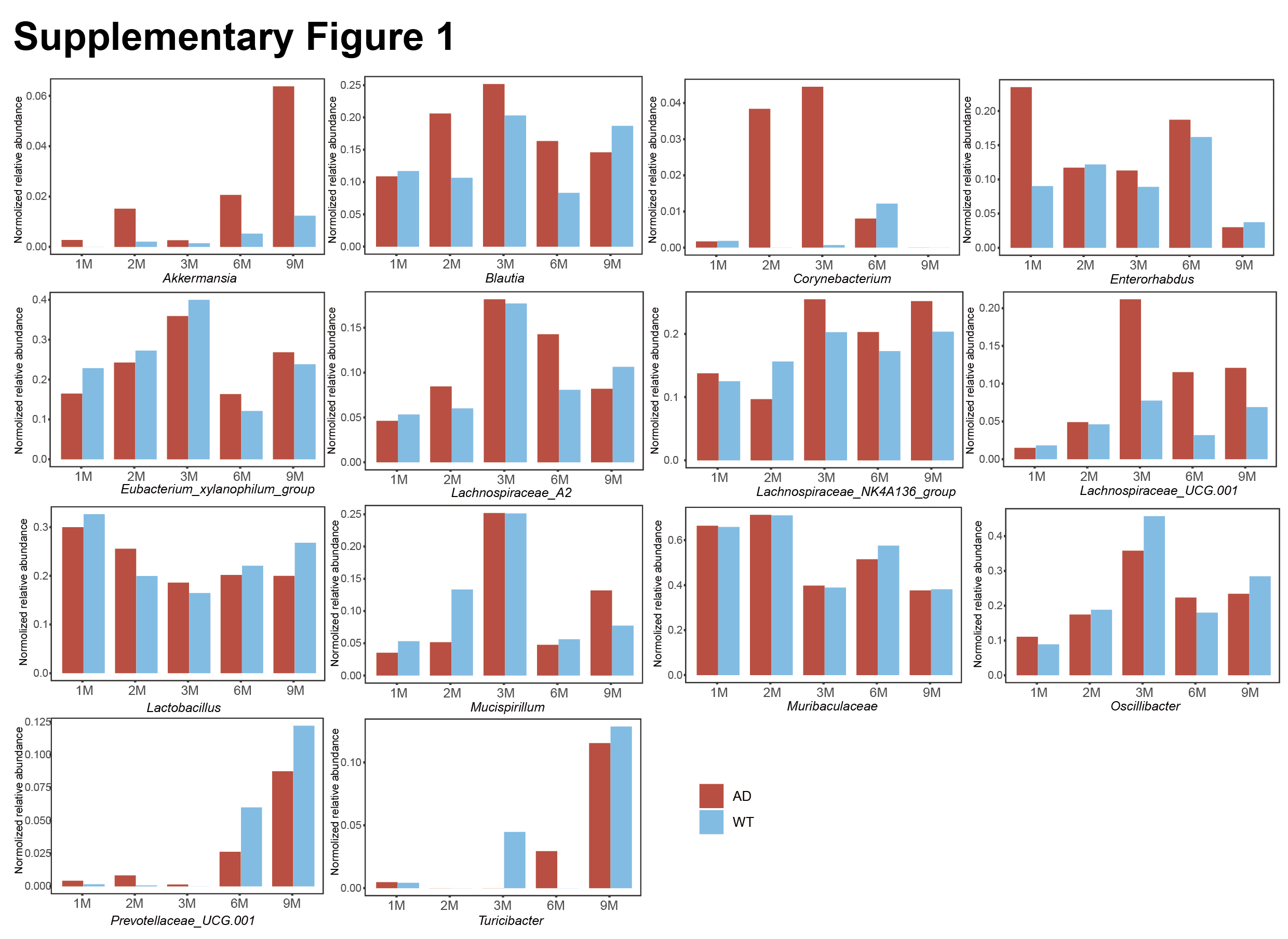

Supplement: Supplemental Material [file KGMI_A_2172672_SM5743.zip › Supplementary Figure 1 (1).jpg]

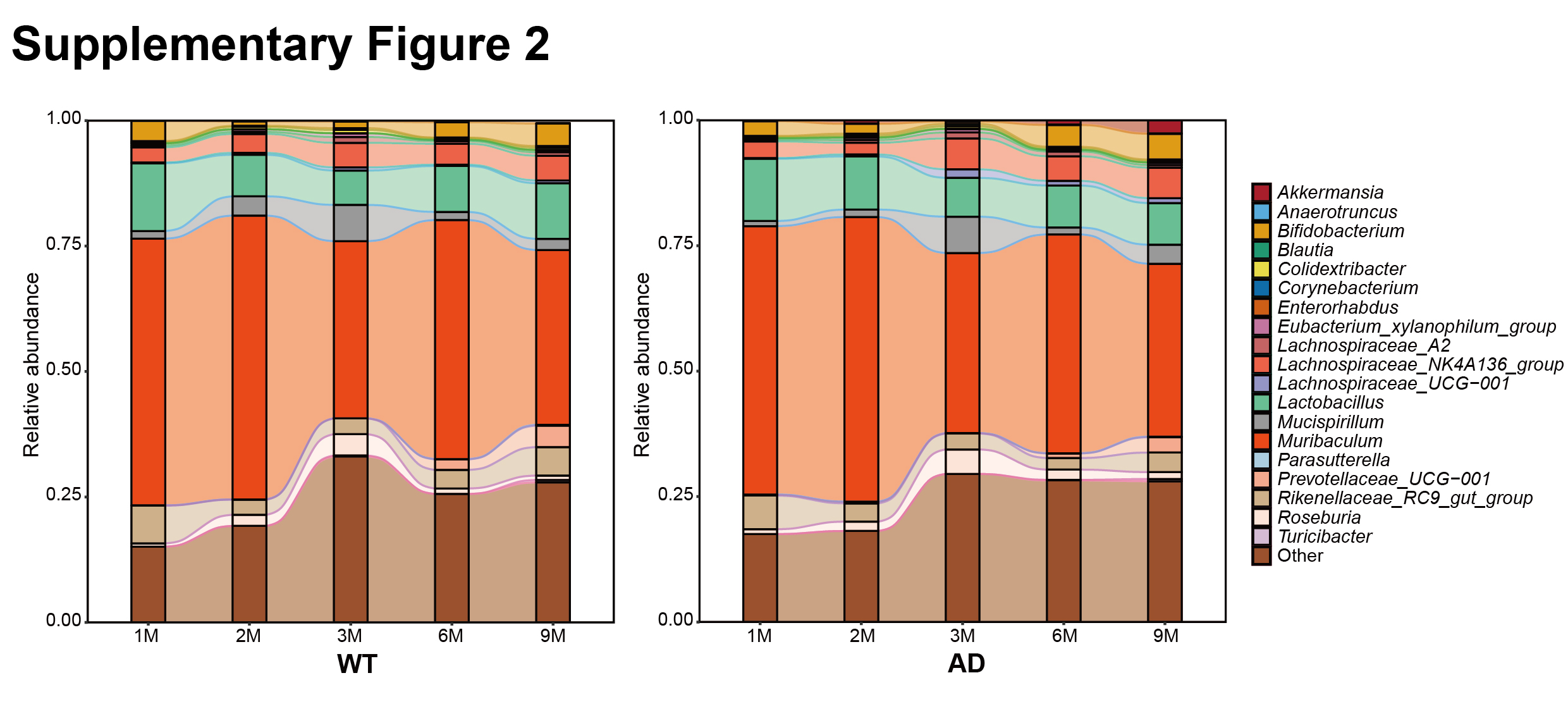

Supplement: Supplemental Material [file KGMI_A_2172672_SM5743.zip › Supplementary Figure 2.jpg]

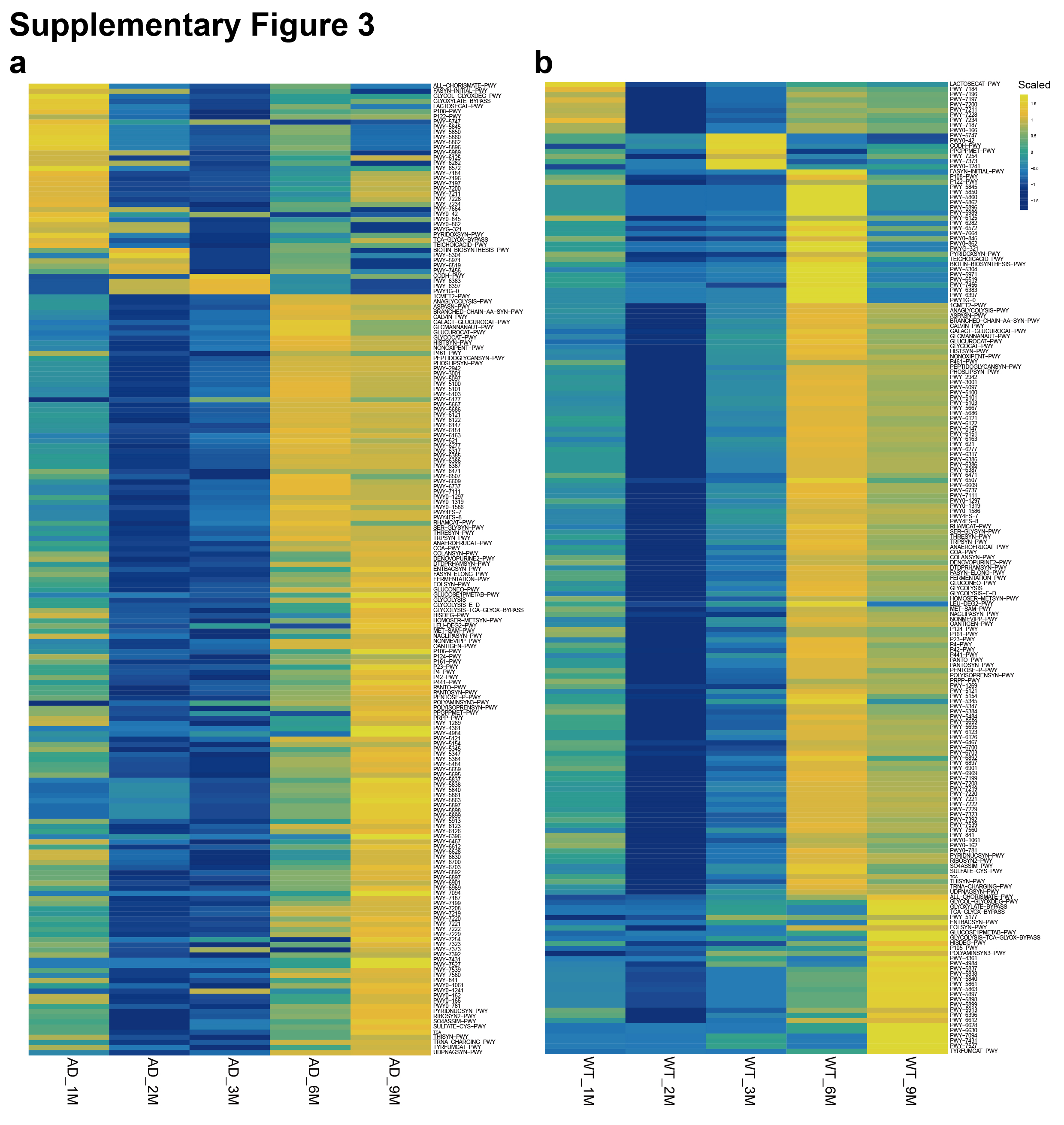

Supplement: Supplemental Material [file KGMI_A_2172672_SM5743.zip › Supplementary Figure 3.jpg]

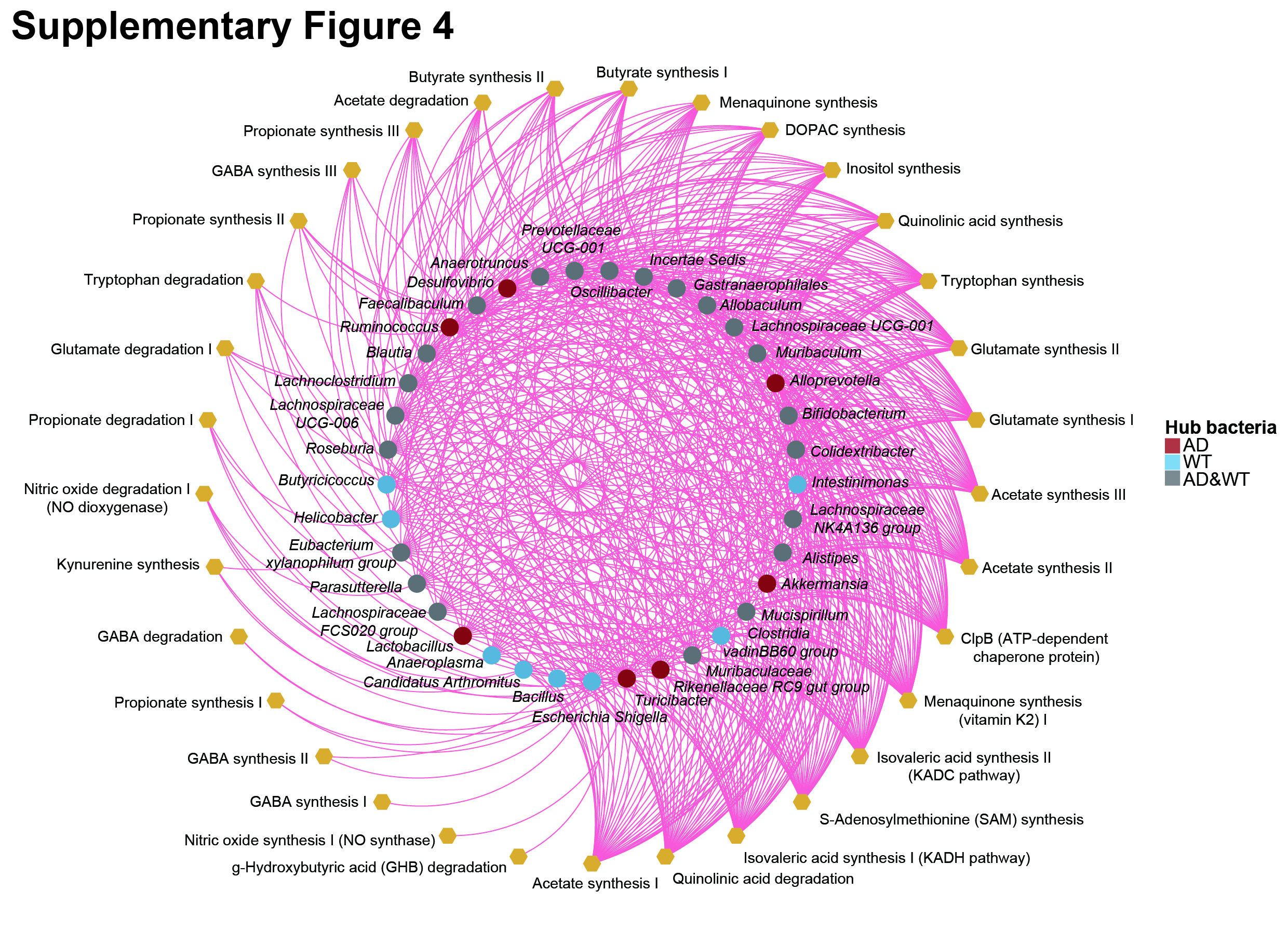

Supplement: Supplemental Material [file KGMI_A_2172672_SM5743.zip › Supplementary Figure 4 (1).jpg]

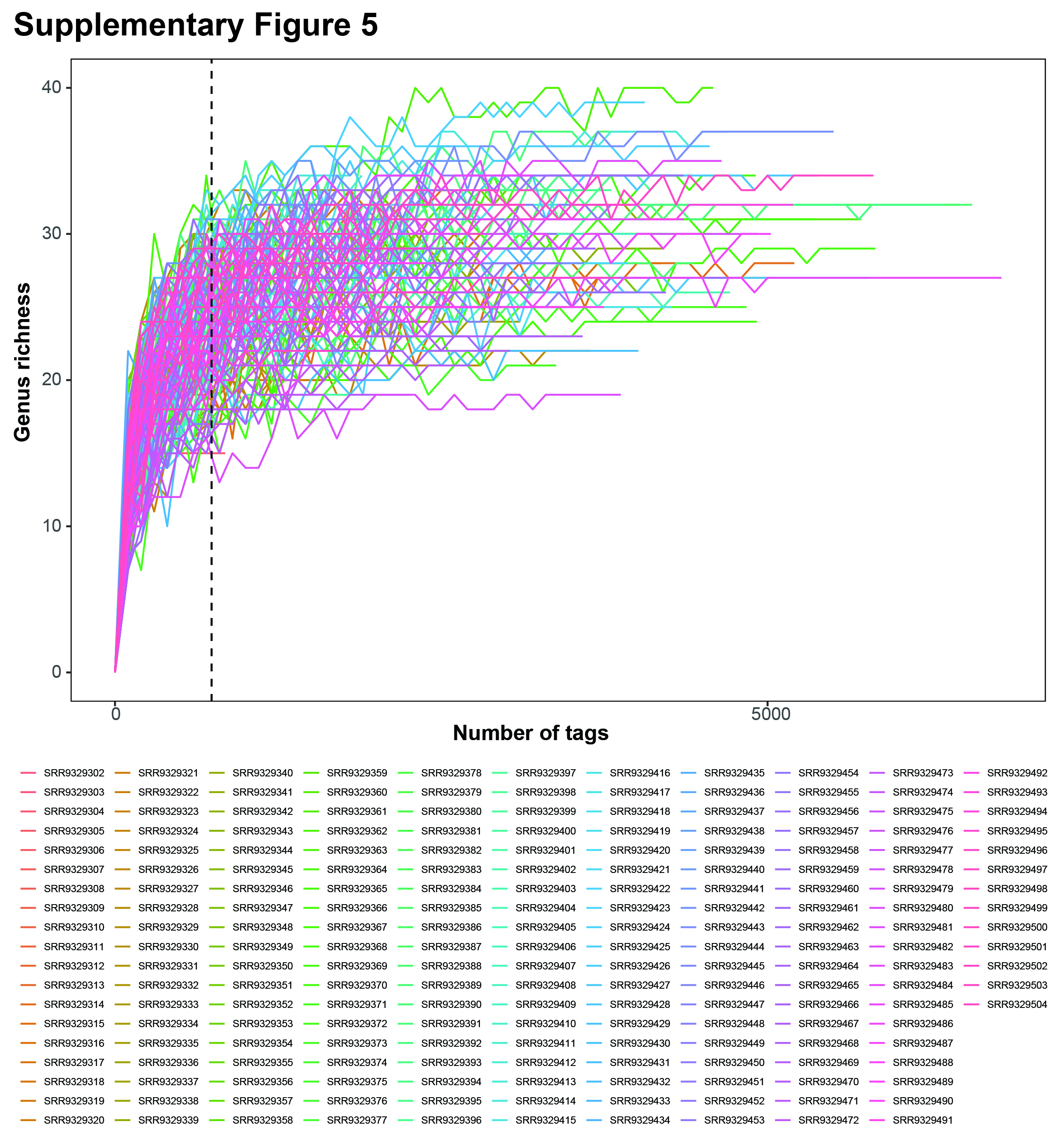

Supplement: Supplemental Material [file KGMI_A_2172672_SM5743.zip › Supplementary Figure 5 (1).jpg]
